# Evaluation Method for Underwater Ultrasonic Energy Radiation Performance Based on the Spatial Distribution Characteristics of Acoustic Power

**DOI:** 10.3390/s24123942

**Published:** 2024-06-18

**Authors:** Zhongzheng Liu, Tao Zhang, Yazhen Yuan, Yuhang Li, Yanzhang Geng

**Affiliations:** School of Electrical and Information Engineering, Tianjin University, Tianjin 300072, China; 1019234089@tju.edu.cn (Z.L.); zhangtao@tju.edu.cn (T.Z.); yuanyazhen@tju.edu.cn (Y.Y.); liyuhang7@tju.edu.cn (Y.L.)

**Keywords:** underwater acoustic wireless energy transmission, acoustic source radiation efficiency, spatial distribution of sound power, assessment of energy radiation performance

## Abstract

In recent years, underwater wireless ultrasonic energy transmission technology (UWUET) has attracted much attention because it utilizes the propagation characteristics of ultrasound in water. Effectively evaluating the performance of underwater ultrasonic wireless energy transmission is a key issue in engineering design. The current approach to performance evaluation is usually based on the system energy transfer efficiency as the main criterion, but this criterion mainly considers the overall energy conversion efficiency between the transmitting end and the receiving end, without an in-depth analysis of the characteristics of the distribution of the underwater acoustic field and the energy loss that occurs during the propagation of acoustic waves. In addition, existing methods focusing on acoustic field analysis tend to concentrate on a single parameter, ignoring the dynamic distribution of acoustic energy in complex aquatic environments, as well as the effects of changes in the underwater environment on acoustic propagation, such as spatial variability in temperature and salinity. These limitations reduce the usefulness and accuracy of models in complex marine environments, which in turn reduces the efficiency of acoustic energy management and optimization. To solve these problems, this study proposes a method to evaluate the performance of underwater ultrasonic energy radiation based on the spatial distribution characteristics of acoustic power. By establishing an acoustic power distribution model in a complex impedance–density aqueous medium and combining numerical simulation and experimental validation, this paper explores the spatial variation of acoustic power and its impact on the energy transfer efficiency in depth. Using high-resolution spatial distribution data and actual environmental parameters, the method significantly improves the accuracy of the assessment and the adaptability of the model in complex underwater environments. The results show that, compared with the traditional method, this method performs better in terms of the accuracy of the acoustic energy radiation calculation results, and is able to reflect the energy distribution and spatial heterogeneity of the acoustic source more comprehensively, which provides an important theoretical basis and practical guidance for the optimal design and performance enhancement of the underwater ultrasonic wireless energy transmission system.

## 1. Introduction

In the process of exploring and utilizing marine resources, the energy supply of underwater equipment is an important technological challenge [1]. In recent years, much attention has been paid to underwater wireless ultrasonic energy transmission (UWUET), a cutting-edge technology which utilizes the properties of ultrasonic waves propagating in water [2,3]. This technology provides a safe, reliable, flexible, and covert charging method for underwater devices by converting electrical energy to acoustic energy and then back to the former [4,5]. Compared with traditional electromagnetic wave wireless energy transmission methods, ultrasonic waves are not affected by electromagnetic interference and eddy current losses and can solve several limitations of electromagnetic induction and magnetic coupling resonant transmission methods for underwater applications, such as the coaxial retention of coils, the magnetic core axis offset, and the instability of the system-coupling state. In addition, acoustic waves have lower propagation losses underwater, resulting in longer propagation distances [6,7,8]. The evaluation of underwater wireless energy transmission performance is of great theoretical and practical significance for system design and optimization [9]. As the complexity of underwater operations increases, traditional methods for evaluating the performance of ultrasonic energy radiation face challenges due to insufficient accuracy and poor adaptability, mainly due to insufficient consideration of the spatial distribution characteristics of acoustic power; this, in turn, affects the efficiency of energy utilization and the overall performance of the system [10,11,12,13,14].

In current research on underwater ultrasonic wireless energy transfer technology, performance evaluation is mainly based on the energy transfer efficiency of the system [15,16]. However, this approach often neglects to conduct detailed analyses from the transmitter to the receiver in complex underwater environments [17]. An ideal evaluation framework should include the electroacoustic conversion efficiency at the transmitting end, the propagation efficiency of sound waves in water, and the acoustic–electric conversion efficiency at the receiving end. Existing evaluations only consider the overall energy conversion efficiency from the transmitting end to the receiving end [18], lack an in-depth analysis of the underwater acoustic field’s distribution and the energy loss during acoustic wave propagation, and fail to adequately consider the complexity of the underwater environment and its impact on acoustic propagation [19]. Moreover, the acoustic energy radiation process occupies an important position in the underwater ultrasonic wireless energy transmission process, which directly affects the energy transmission efficiency and the overall system performance [20]. Therefore, acoustic radiation efficiency is particularly critical in underwater ultrasonic wireless energy transfer technology, and its improvement is one of the key development directions for the latter [21]. Existing evaluation methods focusing on acoustic field analysis usually concentrate on a single parameter, ignoring the dynamic distribution of acoustic energy in complex water [22]. For example, the half-power angle evaluation method is usually used to assess the radiation angle and radiated power of a sound source [23]. The half-power angle corresponds to when the radiated sound power of a sound source in the horizontal or vertical plane is reduced to half of its maximum value and, as such, it can reflect the directionality of the radiation of the sound source: in other words, the smaller the radiation angle of the sound source, the more obvious the directionality and the higher the energy concentration [24]. The advantage of the half-power angle evaluation method lies in its simplicity; however, it fails to fully consider the sound source’s radiation pattern and the radiation field’s distribution characteristics, cannot reflect the inhomogeneity of the radiation of the sound source, and can only calculate the value related to the main lobe [25,26]. Acoustic radiation force evaluation methods suffer from computational difficulties, with controversies and uncertainties regarding the force’s calculation and properties [25]. In addition, acoustic radiation impedance evaluation methods cannot fully consider the radiation shape of the source and the distribution characteristics of the radiation field or adequately, objectively, and accurately assess the radiation performance of underwater ultrasonic energy [19]. These studies rely on propagation models under idealized conditions and fail to reflect the spatial heterogeneity of sound sources in practical applications. In addition, these approaches tend to ignore the effects of underwater environmental changes on acoustic wave propagation, such as spatial variability in temperature and salinity. These shortcomings limit the practicality and accuracy of these models in complex marine environments, thus reducing the efficiency of acoustic energy management and optimization [27,28].

In this study, a high-resolution model for evaluating the performance of underwater ultrasonic energy radiation based on the spatial distribution characteristics of acoustic power in complex impedance density aqueous media is introduced for the first time to address the above problems and simulate and analyze spatial variations in acoustic power and their effect on energy transfer efficiency. By combining sound power spatial distribution data and actual environmental parameters, this method not only improved the assessment accuracy but also enhanced the model’s adaptability in complex underwater environments. First, we derived a mathematical expression for calculating the acoustic energy radiation performance evaluation index and its physical meaning based on the acoustic-radiated power characteristics in complex impedance density media with the multi-parameter effects of an underwater environment. Subsequently, the proposed evaluation method was verified via numerical simulation and an experiment. During the experiments, the acoustic energy radiation performance of arrays processed by several amplitude-weighting algorithms was compared in order to reflect the spatial heterogeneity of the sound sources. The results showed that the method proposed in this study is more accurate in acoustic energy radiation calculation compared to the traditional method. Through an in-depth analysis of the spatial distribution characteristics of acoustic power, this study provides an important theoretical basis and practical guidance for the optimal design and performance enhancement of underwater ultrasonic wireless energy transmission technology.

## 2. Methodology Principle

In this paper, a method for evaluating the performance of underwater ultrasonic energy radiation based on the spatial distribution characteristics of acoustic power is proposed. The spatial distribution characteristic of acoustic power of acoustic radiation refers to the magnitude and distribution of acoustic power radiated into space by an acoustic source under a specific excitation signal [29].

### 2.1. Evaluation Method of Underwater Ultrasonic Energy Radiation Performance Based on Acoustic Power Spatial Distribution Characteristics

#### 2.1.1. Acoustic Wave Propagation Equations in Complex Impedance–Density Media Based on the Multi-Parameter Effects of the Underwater Environment

In underwater environments, the propagation of sound waves is affected by many factors [30]. Acoustic wave propagation in a medium follows the fluctuation equation:

(1) $$\begin{array}{c} {-\frac{1}{\rho_{c}}\nabla }^{2}p_{t}-\frac{K_{eq}^{2}}{\rho_{c}}p_{t}=0 \end{array}$$

This is a fluctuation equation that describes the propagation of sound waves in a medium with a complex impedance density $\rho_{c}$. $p_{t}$ represents the total sound pressure and $K_{eq}$ is the equivalent wavenumber.

Parameter definition:

(2) $$\begin{array}{c} p_{t}=p+p_{b} \end{array}$$

The total sound pressure, $p_{t}$, consists of two components. The sound pressure, p, represents the pressure fluctuation of the sound wave propagating in the medium; the background pressure, $p_{b}$, is the static pressure caused by the environment or other non-acoustic dynamic factors.

(3) $$\begin{array}{c} \rho_{c}=\frac{\rho c^{2}}{{c_{c}}^{2}} \end{array}$$

$\rho_{c}$ represents the complex impedance density of a medium, a parameter used to characterize the impedance of a medium to the propagation of sound waves.

ρ is the density of the medium; this is one of the fundamental physical properties of the medium that affects the speed and behavior of sound waves propagating through the medium.

c is the theoretical speed of sound, which represents the ideal speed of sound without taking into account absorption losses, and is the fundamental speed of sound for wave propagation in a medium, i.e., the speed at which a sound wave propagates in a medium without the influence of other complex factors (e.g., changes in temperature, pressure, etc.). c is the value that represents the speed of sound under standard conditions (reference temperature and pressure).

$c_{c}$ represents the speed of sound under actual conditions. The value of $c_{c}\;$is the speed of sound under actual environmental conditions, incorporating variations due to the non-uniformity of the medium and temperature variations, salinity, acidity, alkalinity, and depth factors.

This equation is essentially a tuning formula which is used to calculate the complex impedance density of a medium under specific conditions. By comparing the theoretical speed of sound, c, and the actual speed of sound, $c_{c}$ squared, it is possible to reflect how the difference between the actual and theoretical speeds of sound affects the impedance characteristics of sound waves in a medium. If $c_{c}$ (actual speed of sound) is lower than c (theoretical speed of sound), then the impedance of the medium to the sound wave increases; complex impedance density, $\rho_{c}$, also increases accordingly, which may be due to the absorption properties of the medium or other factors affecting the speed of sound.

Such calculations are important in acoustic applications, especially when the absorption and impedance properties of the medium need to be accurately taken into account, such as in simulations of hydroacoustic propagation at different water temperatures and depths. Such detailed calculations of complex impedance densities can help to predict and explain the propagation behavior of sound waves in complex environments.

(4) $$\begin{array}{c} K_{eq}^{2}={\left(\frac{\omega }{c_{c}}\right)}^{2} \end{array}$$

$K_{eq}$ is the equivalent wavenumber that characterizes the propagation of sound waves in a medium. ω is the angular frequency, the speed of sound wave vibration.

(5) $$\begin{array}{c} c_{c}=\frac{\omega }{K} \end{array}$$

$c_{c}$ is a specific expression for the speed of sound, related to the angular frequency and the wavenumber *K*. The speed of sound $c_{c}$ is usually affected by temperature, salinity, acidity, and depth, showing a distinctly non-uniform distribution.

(6) $$\begin{array}{c} K=\frac{\omega }{c}-iln\left(10\right)\frac{\alpha }{20}=-\frac{\omega }{c}\cdot \sin\left(\frac{\alpha }{20{}^{\circ}}\right) \end{array}$$

where α is a frequency-dependent parameter characterizing the absorption properties of the medium.

(7) $$\begin{array}{c} \alpha =A_{1}P_{1}\frac{f_{1}f^{2}}{f^{2}+f_{1}^{2}}+A_{2}P_{2}\frac{f_{2}f^{2}}{f^{2}+f_{2}^{2}}+A_{3}P_{3}f^{2} \end{array}$$

(8) $$\begin{array}{c} f_{1}=\left(2.8\;kHz\right){\left(\frac{S_{p}}{35}\right)}^{0.5}\times 10^{4\cdot \frac{\left(1245K\right)}{T}} \end{array}$$

(9) $$\begin{array}{c} f_{2}=\frac{\left(8.17\;kHz\right)\times 10^{8\cdot \frac{\left(1990K\right)}{T}}}{1+0.0018\left(S_{p}-35\right)} \end{array}$$

These are related to the temperature- (T) and salinity ($S_{p}$)-related frequency tuning parameter formulas.

(10) $$\begin{array}{c} P_{2}=1-\left(1.37\times 10^{-4}m^{-1}\right)D+\left(6.2\times 10^{-9}m^{-2}\right)D^{2} \end{array}$$

(11) $$\begin{array}{c} P_{3}=1-\left(3.83\times 10^{-5}m^{-1}\right)D+\left(4.9\times 10^{-10}m^{-2}\right)D^{2} \end{array}$$

(12) $$\begin{array}{c} A_{1}=\frac{8.86\;m/s}{c}\times 10^{0.78pH-5} \end{array}$$

(13) $$\begin{array}{c} A_{2}=\frac{\left(21.44\;m/s\right)S_{p}}{c}\left(1+\left(0.025K^{-1}\right)\left(T-T_{ref}\right)\right) \end{array}$$

(14) $$\begin{array}{c} A_{3}=\left\{\begin{array}{c} 4.937\times 10^{-4}-\left(2.59\times 10^{-5}K^{-1}\right)\left(T-T_{ref}\right)+\left(9.11\times 10^{-7}K^{-2}\right){\left(T-T_{ref}\right)}^{2}-\left(1.50\times 10^{-8}K^{-3}\right){\left(T-T_{ref}\right)}^{3}\;T\leq 20\;{}^{\circ}C\\ 3.964\times 10^{-4}-\left(1.146\times 10^{-5}K^{-1}\right)\left(T-T_{ref}\right)+\left(1.45\times 10^{-7}K^{-2}\right){\left(T-T_{ref}\right)}^{2}-\left(6.5\times 10^{-10}K^{-3}\right){\left(T-T_{ref}\right)}^{3}\;T\geq 20\;{}^{\circ}C \end{array}\right. \end{array}$$

These parameters are related to the medium temperature, *T*, the salinity$,\;S_{p}$, the acidity, *pH*, and the pressure, *D*. They are used to calculate absorption coefficients and related influence factors in sound wave propagation. The effect of temperature on the speed of sound is described by a series of complex polynomial expressions divided into different temperature intervals. These expressions take into account the temperature, *T*, and the reference temperature, $T_{ref}$, differences and are used to accurately calculate the change in the speed of sound under different conditions.

These equations and parameters are particularly important in calculating acoustic wave propagation in aqueous media, especially under changing environmental conditions (e.g., temperature, salinity, pH, and depth variations). These equations integrate complex relationships between multiple variables and, within certain limits, can more accurately predict the behavior of sound waves in real-world environments. In practical applications, sound velocity profiles are commonly used to determine the path and refraction properties of sound wave propagation. On this basis, a ray model applicable to underwater ultrasonic wave propagation can be constructed, and the sound field distribution can be determined by tracing the path of acoustic rays.

#### 2.1.2. Sound Field Distribution Model

Equation (1) for acoustic wave propagation in complex impedance–density media based on the multi-parameter effects of the underwater environment given above, which is essentially a deformation of the fluctuation equation, is used to solve for the distribution of sound pressure in the medium for a given frequency and speed of sound.

Further simplification of Equation (1) yields:

(15) $$\begin{array}{c} \nabla^{2}p_{t}+K_{eq}^{2}p_{t}=0 \end{array}$$

This is a classical form of the Helmholtz equation, the solution of which depends on specific boundary and initial conditions [31]. In this paper, we investigate the modeling of acoustic field distribution describing underwater ultrasonic radiation in an open three-dimensional free space. That is, in the three-dimensional case, based on the Neumann boundary conditions, the solution of the equation can be expressed as:

(16) $$\begin{array}{c} p_{t}=Ae^{i\left(K_{eq}\vec{\cdot r}-\omega t\right)} \end{array}$$

where $\vec{r}$ is the position from the sound source; *t* is the time; *A* is the amplitude, which is determined by boundary conditions or initial conditions. The sound pressure is known at the boundary *x* = 0, i.e., $p_{t}\left(0,t\right)=p_{0}(t)$, and this condition is substituted into the waveform equation to solve for *A*.

#### 2.1.3. Sound Power Calculation Method

Sound power calculations are central to evaluating the performance of underwater ultrasonic energy radiation. Sound power is the rate of transmission of acoustic energy and can be calculated by evaluating the amount of acoustic energy passing through a given area per unit of time [32].

Sound intensity, *I*, is the sound energy flowing per unit area, per unit time, defined as the time average of the instantaneous product of the sound pressure, *p*, and the sound particle velocity, *v*:

(17) $$\begin{array}{c} I=\frac{1}{2}Re\left(\rho v^{*}\right) \end{array}$$

where $v^{*}$ denotes the complex conjugate of *v*.

The acoustic particle velocity *v* can be calculated from the derivative of the acoustic pressure with respect to the density of the medium *ρ*:

(18) $$v=\frac{1}{j\omega \rho }\nabla p$$

The acoustic power, *W*, can be obtained by integrating the acoustic intensity, *I*, over a specific closed surface, *S*:

(19) $$W=\int_{S}I\cdot dS=\int_{S}\left(\frac{1}{2}Re\left(\rho_{c}{\left(\frac{1}{j\omega \rho_{c}}\nabla p_{t}\right)}^{*}\right)\right)\cdot dS\;$$

Here, *dS* is a vector of surface elements pointing to the outside of the closed surface.

#### 2.1.4. Sound Power in the Effective Radiant Area

Calculating the sound power at a specific direction angle and distance requires choosing an appropriate control surface, which can be a very small spherical area (or a small surface element in a specific direction). We need to define this particular area element, *dS*, and the projection of the acoustic intensity, *I*, on that surface.

Defining the area microelement *d*S.

Effective radiated area *dS* is the area of the region after the sound source energy is radiated to a certain distance according to a specific angle. It reflects the actual coverage of the sound source energy in the target area.

**Figure 1:**
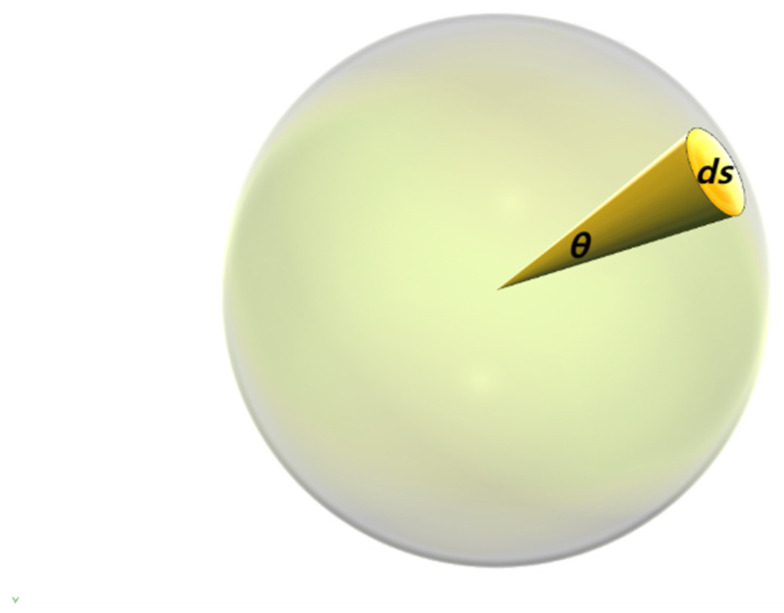
Acoustic power sphere closed surface, *S*.

As shown in Figure 1, the closed surface, S, enclosing the sound source is a sphere of arbitrary radius centered on the sound source. The closed surface, *S*, is a sphere with radius, *r*, and area, $4\pi r^{2}$. The area element *dS* at a particular cone angle, *θ*, can be expressed as

(20) $$\begin{array}{c} dS=r^{2}\sin\left(\theta \right)d\theta \end{array}$$

Here, the acoustic-radiated power angle, *θ*, is the angle from the sound source to the observation point. On the closed surface, *S*, the actual coverage of acoustic energy in the target area can be calculated from the area element *d*S.

Calculate the effective radiated area sound power:

According to Formula (19), the acoustic power *W* is given by the integration of the acoustic intensity, *I*, over the area element *d*S, the exact calculation of which depends on the magnitude of *I* in that direction.

In practical acoustic applications, it may be necessary to integrate the entire closed surface or a portion of a region in a specific direction to obtain the total sound power or the sound power in a specific direction. The exact range of integration depends on the characteristics of the source and the location of the receiving point.

The effective radiated area sound power can be calculated by substituting a specific direction angle and distance into the area element ds:

(21) $$\begin{array}{c} W_{r}=I\cdot r^{2}\sin\left(\theta \right)d\theta \end{array}$$

To calculate the total power radiated by the energy of the source, i.e., the sound intensity *I* does not vary with the angle of the acoustic-radiated power *θ*, the total acoustic power over the whole closed sphere *S* is:

(22) $$\begin{array}{c} W_{t}=\int_{0}^{2\pi }I\cdot r^{2}\sin\left(\theta \right)d\theta =I\cdot 4\pi r^{2} \end{array}$$

The effective radiated area sound power is calculated by setting the area element *d*S according to a specific direction angle, distance $W_{r}$, total power radiated by the sound source energy $W_{t}$, and total power radiated by the sound source. This method can reflect the density of the energy radiation distribution of the sound source in local details and in the whole, and the unevenness of the sound field distribution is fully considered in the calculation, so that a more accurate value of the acoustic energy radiation power can be obtained.

#### 2.1.5. Acoustic Energy Radiation Efficiency

In order to evaluate the performance of underwater ultrasonic energy radiation based on the acoustic power characteristics of acoustic radiation, we need to establish the underwater ultrasonic energy radiation efficiency function.

(23) $$\begin{array}{c} \eta =\frac{W_{r}}{W_{t}}=\frac{Ir^{2}\sin\left(\theta \right)d\theta }{\int_{0}^{2\pi }I\cdot r^{2}\sin\left(\theta \right)d\theta } \end{array}$$

Here$,\;W_{r}$ represents the acoustic radiated energy power projected onto the effective radiated area ds at a specific direction angle and distance, and $W_{t}$ represents the total energy radiated power of the acoustic field. The parameters defined in this paper η can be used to characterize the distribution of acoustic energy in a specific direction, which can measure the uniformity of energy radiation and energy concentration of the sound source and are important for analyzing and optimizing the directionality and focalization of the sound source.

### 2.2. Evaluation Method of Acoustic Energy Radiation Performance Based on Ultrasonic Transducer Array Modeling

To establish a mathematical model of acoustic energy radiation from an underwater ultrasonic transducer array, we need to consider the physical configuration of the transducer and the propagation characteristics of sound waves in water. The following subsections detail the derivation steps and the equations needed.

#### 2.2.1. Vibration Modeling of the Piezoelectric Transducer

Define the following parameters:

An ultrasonic transducer array is composed of *N* identical circular piezoelectric transducer units, each with radius *a*, thickness *h*, array element spacing *d*, and a material with piezoelectric strain coefficient of${\;d}_{33}$. Assuming that the excitation signal of the ultrasonic transducer array is a single-frequency sinusoidal wave with frequency *f* and angular frequency ω *=2*π*f*. A linear array is selected for this study. The purpose of this study is to evaluate the energy radiation performance of the sound source, the array setup parameters are for reference only, and the replacement of other parameters does not affect the performance evaluation method.

Under voltage excitation, a piezoelectric transducer converts an electrical signal into a mechanical vibration and produces a corresponding sound pressure [33]. Assuming that the piezoelectric transducer is excited by a voltage, *V*, its piezoelectric effect leads to a strain, *u*, in the material. The strain is a deformation induced by an electric field and can be expressed by the piezoelectric constant $d_{33}$ which can be expressed by the piezoelectric constant with the following equation:

(24) $$\begin{array}{c} u=d_{33}\frac{V}{h} \end{array}$$

#### 2.2.2. Sound Pressure of the Transducer

Due to the deformation *u* of the piezoelectric material, mechanical vibrations will be generated on the surface of the transducer, and this vibration will propagate in the medium to form sound waves [34]. The acoustic field generated by a single transducer can be solved by the wave equation. In this study acoustic radiation from an ultrasonic transducer array is a far-field problem, i.e., the observation point is located at a distance much greater than the wavelength from the ultrasonic transducer array. The acoustic pressure, *p*, is given by the following equation:

(25) $$\begin{array}{c} p=Ae^{i\left(K_{eq}R-\omega t\right)}=\frac{ua^{2}\rho_{c}c_{c}\omega^{2}}{2R}e^{i\left(K_{eq}R-\omega t\right)} \end{array}$$

where $\rho_{c}$ is the complex impedance density of the aqueous medium,${\;c}_{c}$ is the speed of sound under actual environmental conditions, *ω* is the angular frequency of the sound wave, $K_{eq}$ is the equivalent wave number, *R* is the distance between the observation point and the center of the transducer, and *t* is the time variable.

The amplitude, A, of the sound pressure is determined by the vibrational displacement *u* of the transducer, the geometry of the transducer (radius *a*), the physical properties of the medium (density and speed of sound), and the frequency of the sound wave, ω.

By calculating an expression for sound pressure based on physical parameters, we can quantify how piezoelectric effects affect the distribution of the sound field.

#### 2.2.3. Sound Pressure of the Array

For a linear array of *N* transducers, the total sound pressure is the superposition of the individual transducer sound pressures [35], and the total sound pressure *P* is given by the following equation:

(26) $$\begin{array}{c} P=\frac{ua^{2}\rho_{c}c_{c}\omega^{2}}{2}\sum_{n=1}^{N}\frac{e^{i\left(K_{eq}R_{n}-\omega t\right)}}{R_{n}} \end{array}$$

where $R_{n}$ is the distance from the center of the *n*th transducer to the observation point.

Due to the mutual interference between the array elements, the calculation of the actual sound field needs to take into account the directionality and focusing effect of the array. The array factor *AF* is usually given by the following equation:

(27) $$\begin{array}{c} AF=\frac{\sin\left(Nkd\cos\left(\theta \right)∕2\right)}{N\sin\left(kd\cos\left(\theta \right)∕2\right)} \end{array}$$

where *θ* is the angle between the viewing direction and the array normal.

The array factor *AF* is obtained by incorporating it into the total sound pressure expression:

(28) $$\begin{array}{c} P=\frac{ua^{2}\rho_{c}c_{c}\omega^{2}}{2}\left(\frac{\sin\left(NK_{eq}d\cos\left(\theta \right)∕2\right)}{N\sin\left(K_{eq}d\cos\left(\theta \right)∕2\right)}\right)\sum_{n=1}^{N}\frac{e^{i\left(K_{eq}R_{n}-\omega t\right)}}{R_{n}} \end{array}$$

#### 2.2.4. Calculation of Sound Power

The sound intensity *I* is the flow of acoustic energy per unit area and is usually expressed as the average of the product of the real part of the complex conjugate of the sound pressure, *p*, and the particle velocity, *v*. The particle velocity can be obtained from the following relation:

(29) $$\begin{array}{c} v=\frac{p}{\rho c} \end{array}$$

Replacing *p* with the sound pressure equation we derived earlier gives us

(30) $$\begin{array}{c} v=\frac{ua^{2}\rho_{c}c_{c}\omega^{2}}{2\rho_{c}c_{c}}\left(\frac{\sin\left(NK_{eq}d\cos\left(\theta \right)∕2\right)}{N\sin\left(K_{eq}d\cos\left(\theta \right)∕2\right)}\right)\sum_{n=1}^{N}\frac{e^{i\left(K_{eq}R_{n}-\omega t\right)}}{R_{n}} \end{array}$$

Substitute *v* into the expression of sound intensity *I* derived above (17):

(31) $$\begin{array}{c} I=\frac{1}{2}\left|\frac{ua^{2}\omega^{2}}{2}\left(\frac{\sin\left(NK_{eq}d\cos\left(\theta \right)∕2\right)}{N\sin\left(K_{eq}d\cos\left(\theta \right)∕2\right)}\right)\sum_{n=1}^{N}\frac{1}{{R_{n}}^{2}}\right| \end{array}$$

Substituting the sound intensity *I* into Equation (20) the acoustic power *W* of the underwater ultrasonic transducer array can be expressed as:

(32) $$W=\int_{S}\frac{1}{2}\left|\frac{ua^{2}\omega^{2}}{2}\left(\frac{\sin\left(NK_{eq}d\cos\left(\theta \right)∕2\right)}{N\sin\left(K_{eq}d\cos\left(\theta \right)∕2\right)}\right)\sum_{n=1}^{N}\frac{1}{{R_{n}}^{2}}\right|dS$$

where *S* is a closed surface around the transducer and *d*S is a surface element. For a spherical surface,

(33) $$\begin{array}{c} dS=R^{2}\sin\left(\theta \right)d\theta \end{array}$$

The effective radiated area acoustic power of the underwater ultrasonic transducer array can be calculated according to Equation (22):

(34) $$\begin{array}{c} W_{r}=\frac{1}{2}\left|\frac{ua^{2}\omega^{2}}{2}\left(\frac{\sin\left(NK_{eq}d\cos\left(\theta \right)∕2\right)}{N\sin\left(K_{eq}d\cos\left(\theta \right)∕2\right)}\right)\sum_{n=1}^{N}\frac{1}{{R_{n}}^{2}}\right|\cdot R^{2}\sin\left(\theta \right)d\theta \end{array}$$

The total energy radiated power of the underwater ultrasonic transducer array can be calculated according to Equation (23):

(35) $$\begin{array}{c} W_{t}=\int_{0}^{2\pi }\frac{1}{2}\left|\frac{ua^{2}\omega^{2}}{2}\left(\frac{\sin\left(NK_{eq}d\cos\left(\theta \right)∕2\right)}{N\sin\left(K_{eq}d\cos\left(\theta \right)∕2\right)}\right)\sum_{n=1}^{N}\frac{1}{{R_{n}}^{2}}\right|\cdot R^{2}\sin\left(\theta \right)d\theta \end{array}$$

The total energy radiated power of the underwater ultrasonic transducer array can be simplified as:

(36) $$\begin{array}{c} W_{t}=\int_{0}^{2\pi }\left|\frac{ua^{2}\omega^{2}}{4}{\left(\frac{\sin\left(NK_{eq}d\cos\left(\theta \right)∕2\right)}{N\sin\left(K_{eq}d\cos\left(\theta \right)∕2\right)}\right)}^{2}\sum_{n=1}^{N}\frac{1}{{R_{n}}^{2}}\right|\cdot R^{2}\sin\left(\theta \right)d\theta \end{array}$$

$$W_{t}=\left(\frac{ua^{2}\omega^{2}}{4}\right)\left(\sum_{n=1}^{N}1\right)\int_{0}^{2\pi }{\left(\frac{\sin\left(NK_{eq}d\cos\left(\theta \right)∕2\right)}{N\sin\left(K_{eq}d\cos\left(\theta \right)∕2\right)}\right)}^{2}\sin\left(\theta \right)d\theta$$

#### 2.2.5. Acoustic Radiation Efficiency of the Underwater Ultrasonic Transducer Array

Based on our establishment of the underwater ultrasonic energy radiation efficiency function (24), the acoustic energy radiation performance of underwater ultrasonic transducer arrays based on the spatial distribution characteristics of acoustic power can be evaluated:

(37) $$\begin{array}{c} \eta =\frac{W_{r}}{W_{t}}=\frac{\frac{1}{2}\left|\frac{ua^{2}\omega^{2}}{2}\left(\frac{\sin\left(NK_{eq}d\cos\left(\theta \right)∕2\right)}{N\sin\left(K_{eq}d\cos\left(\theta \right)∕2\right)}\right)\sum_{n=1}^{N}\frac{1}{{R_{n}}^{2}}\right|\cdot R^{2}\sin\left(\theta \right)d\theta }{\int_{0}^{2\pi }\left|\frac{ua^{2}\omega^{2}}{4}{\left(\frac{\sin\left(NK_{eq}d\cos\left(\theta \right)∕2\right)}{N\sin\left(K_{eq}d\cos\left(\theta \right)∕2\right)}\right)}^{2}\sum_{n=1}^{N}\frac{1}{{R_{n}}^{2}}\right|\cdot R^{2}\sin\left(\theta \right)d\theta } \end{array}$$

The equation for the acoustic energy radiation efficiency of an underwater ultrasonic transducer array can be simplified as:

(38) $$\begin{array}{c} \eta =\frac{\int_{0}^{\pi }{\left(\frac{\sin\left(NK_{eq}d\cos\left(\theta \right)/2\right)}{N\sin\left(K_{eq}d\cos\left(\theta \right)/2\right)}\right)}^{2}\sin\left(\theta \right)d\theta }{2} \end{array}$$

Here$,\;W_{r}$ represents the acoustic radiated energy power projected onto the effective radiating area ds in the receiving direction, and$\;W_{t}$ represents the total energy radiated power of the sound field.

## 3. Design of the Experimental Simulation Model

To validate the effectiveness of the method proposed in this research and compare it to existing acoustic radiation energy analysis methods, we set the experimental environment as infinite-space water and established an open square space with a volume of $V_{water}$. A single small-size transducer tends to produce an omnidirectional sound field, with the sound energy uniformly distributed in all directions. To further validate the ability of the method proposed in this study to analyze the radiation shape and field distribution characteristics of sound sources against conventional methods, we designed an experiment using a transducer array reflecting spatial heterogeneity as a sound source [36]. The purpose of this experiment was to evaluate the energy radiation performance of the sound source, so the array setup parameters were for reference only and replacing other parameters would not affect the validity of the performance evaluation method. Linear arrays were chosen as the object of this study.

**Figure 2:**
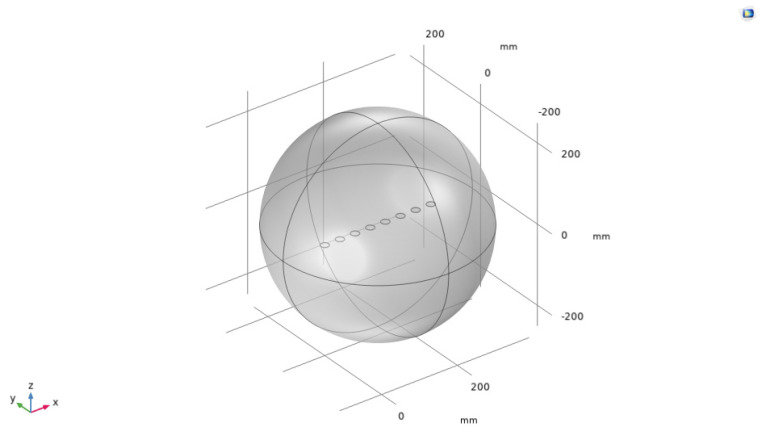
Acoustic power sphere encircling the underwater ultrasonic transducer array.

**Figure 3:**
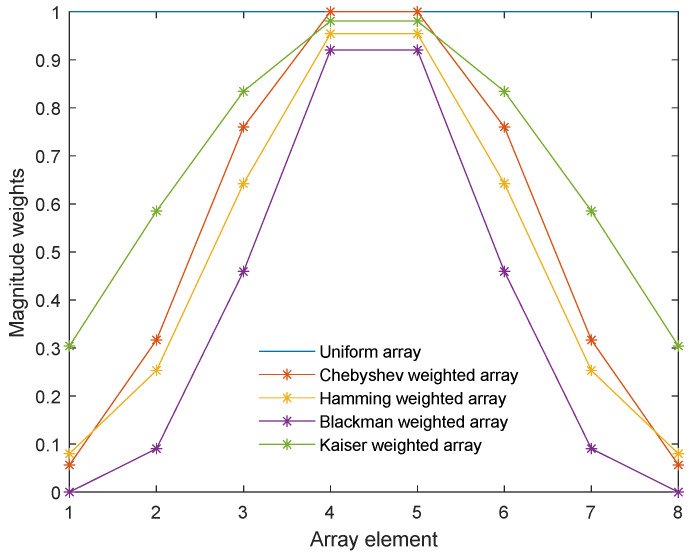
Weights for different arrays.

As shown in Figure 2, the x–y–z axes represented the spatial coordinates, and a linear array with *N* array elements was set up in the space, consisting of circular piezoelectric ceramic sheets with radius a and thickness d, made of the commonly used PZT-4 material, and the array element spacing d was in the half-wavelength range [37]. Since the higher the frequency of the sound wave, the greater the attenuation in water, a frequency of 16 kHz, commonly used in practical applications, was set as the experimental condition, and the model was subjected to an excitation electric signal with rated voltage $V_{rms}$ [38]. In this study, several weighted arrays, commonly used to create a low side-lobe-level beam, were selected as a comparison for the experiment [39], whose array weight settings are shown in Figure 3.

**Figure 4:**
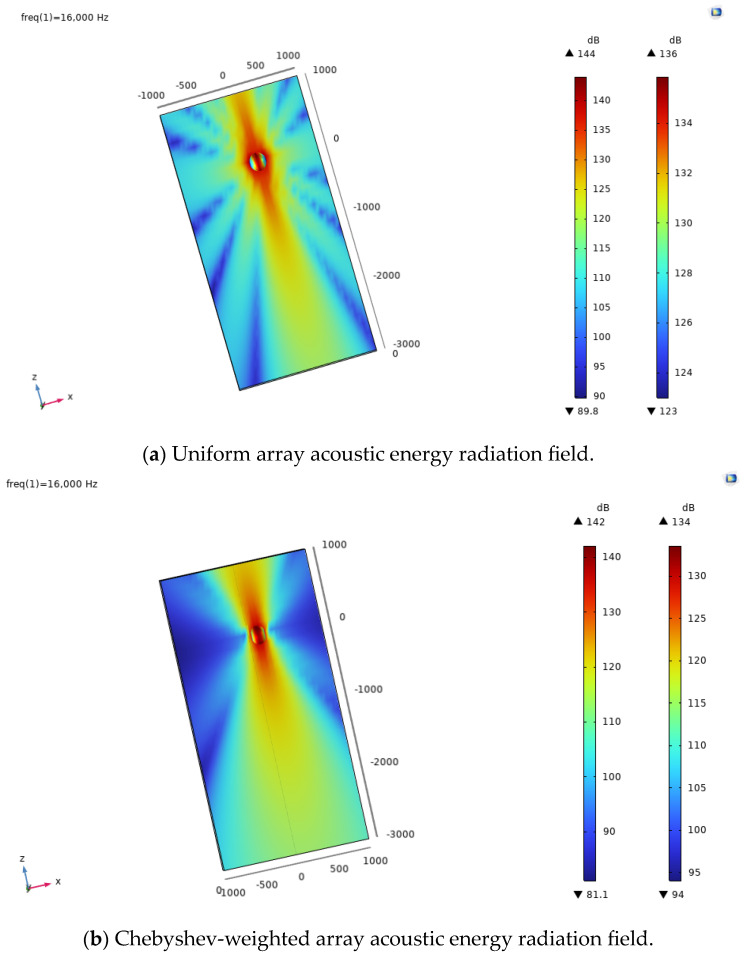

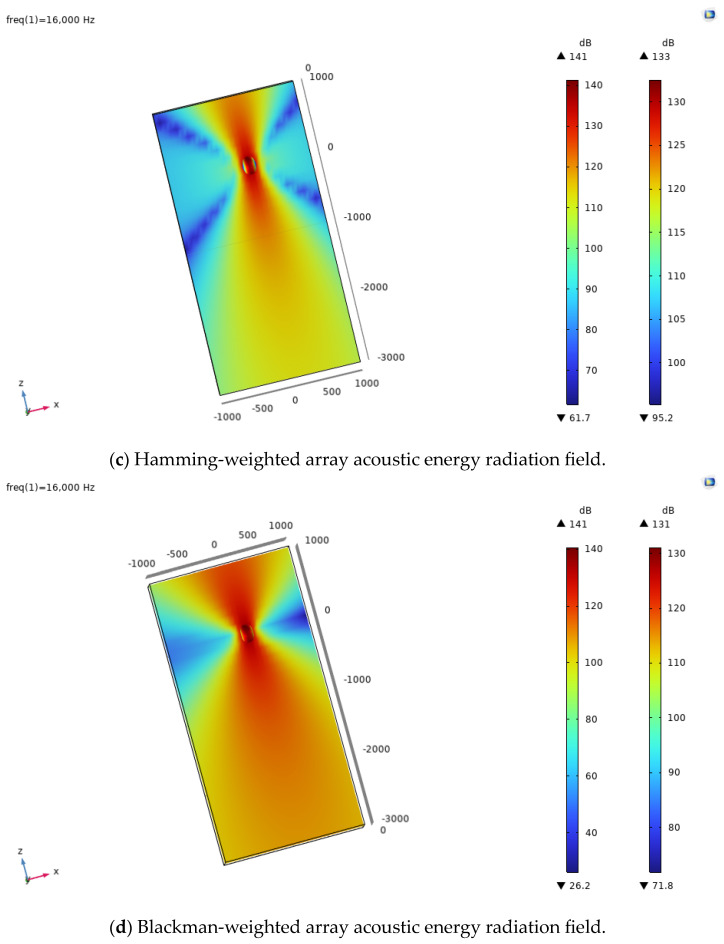

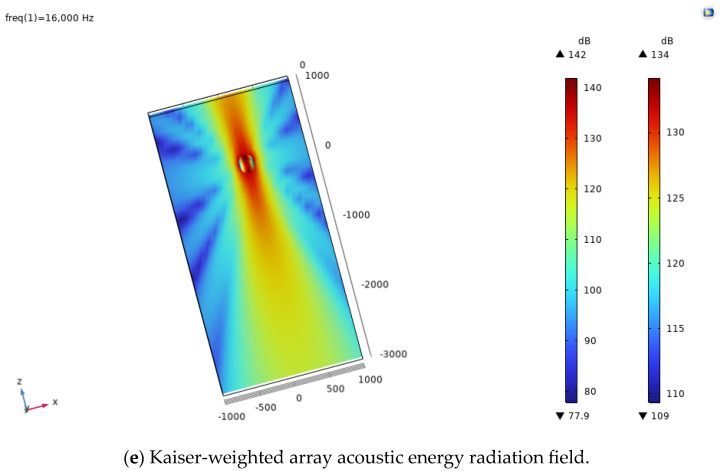
Underwater ultrasonic transducer array acoustic energy radiation fields.

**Figure 5:**
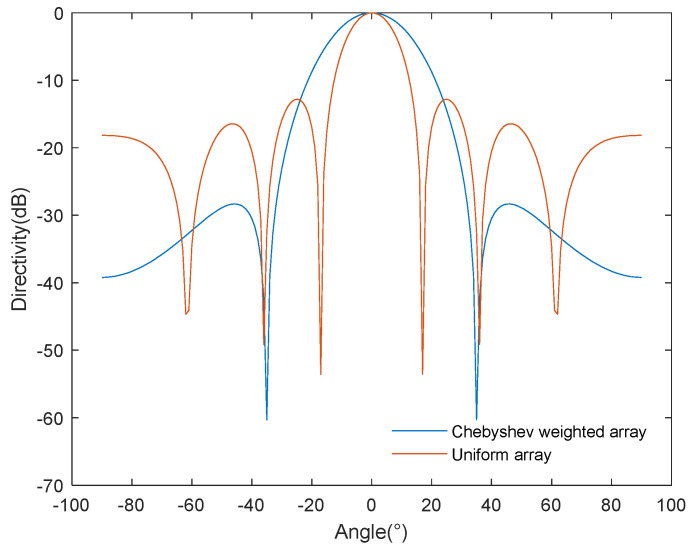
Comparison of the directivity between the uniform ultrasonic transducer array and the Chebyshev-weighted array obtained using the half-power angle method.

**Figure 6:**
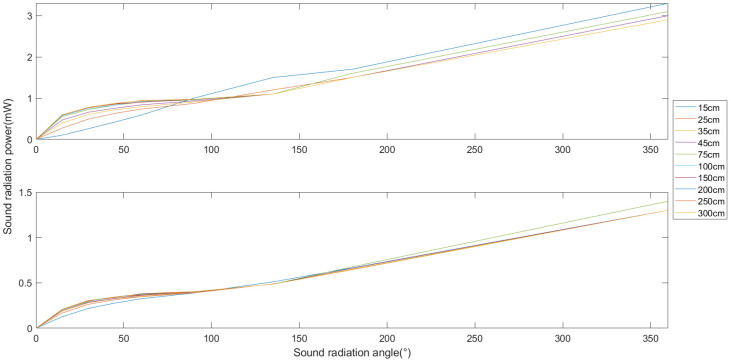
Comparison of the acoustic radiation power calculated using the proposed method between a uniform ultrasonic transducer array and a Chebyshev-weighted array.

**Figure 7:**
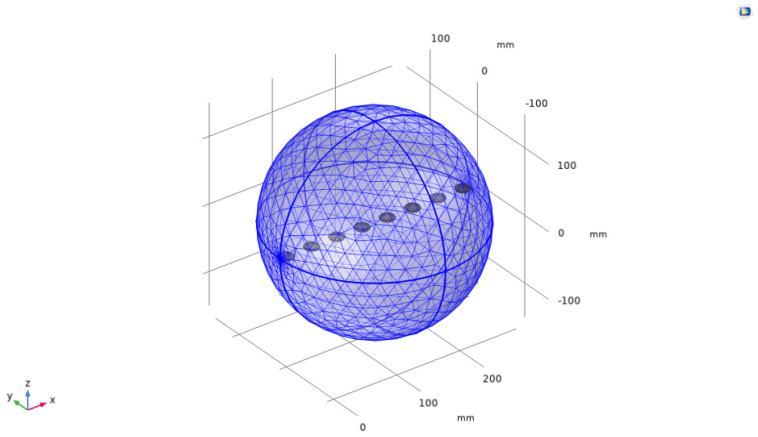
Sound power analysis based on high-resolution spatial distribution model.

**Figure 8:**
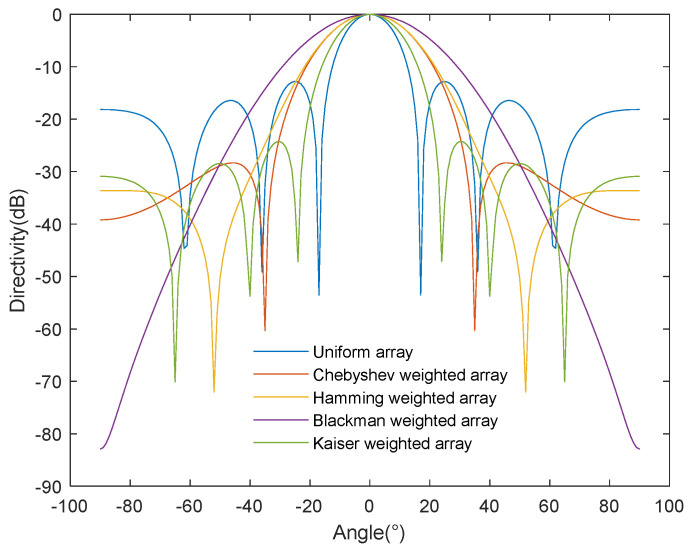
Comparison of the directivity of different arrays obtained using the half-power beamwidth method.

**Figure 9:**
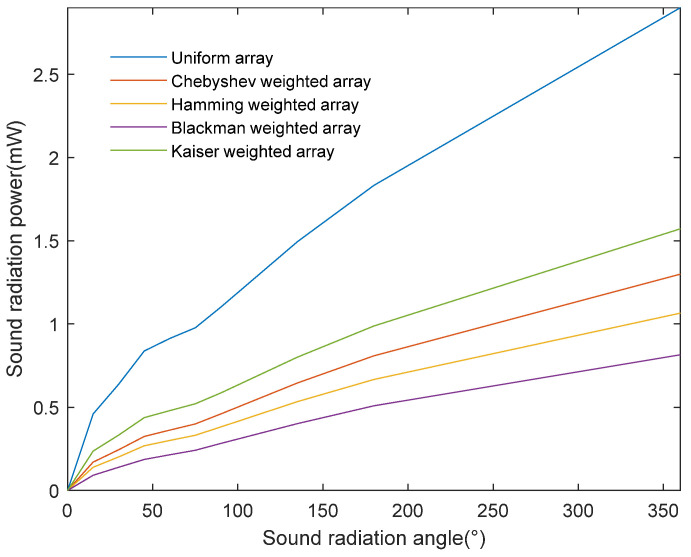
Comparison results of the radiated power of different arrays with a 300 cm acoustic radiation radius calculated using the proposed method.

**Figure 10:**
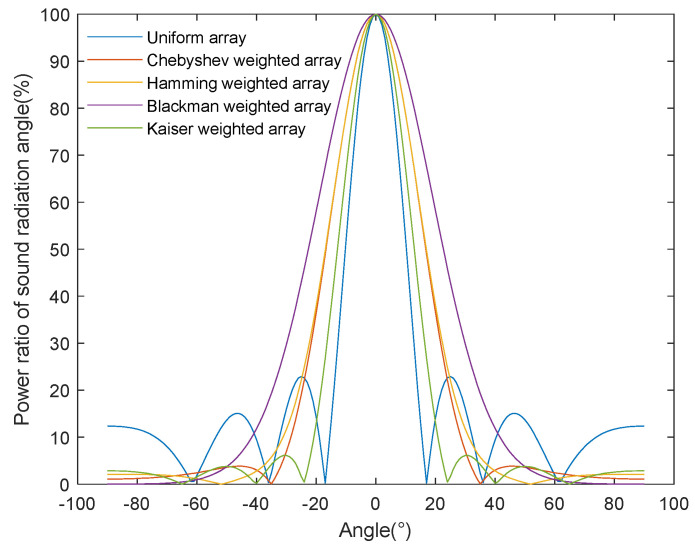
Comparison results of the array energy radiation percentage for different acoustic-radiated power angles calculated using the half-power beamwidth method.

**Figure 11:**
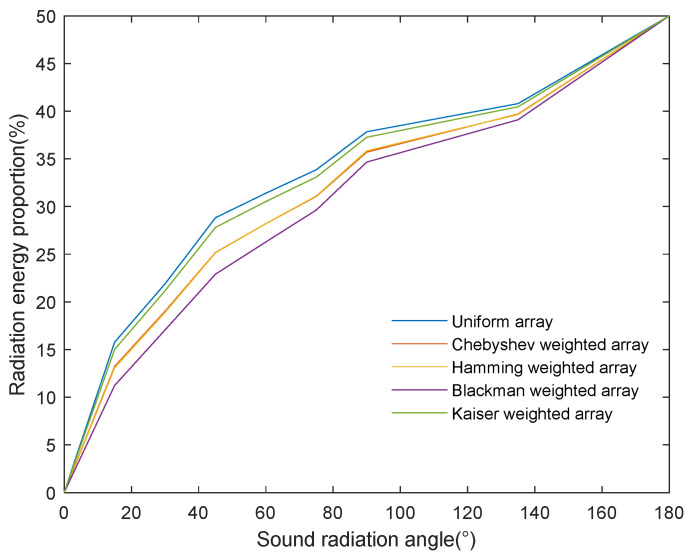
Comparison results of the acoustic radiation efficiency of different arrays calculated using the proposed method.

**Figure 12:**
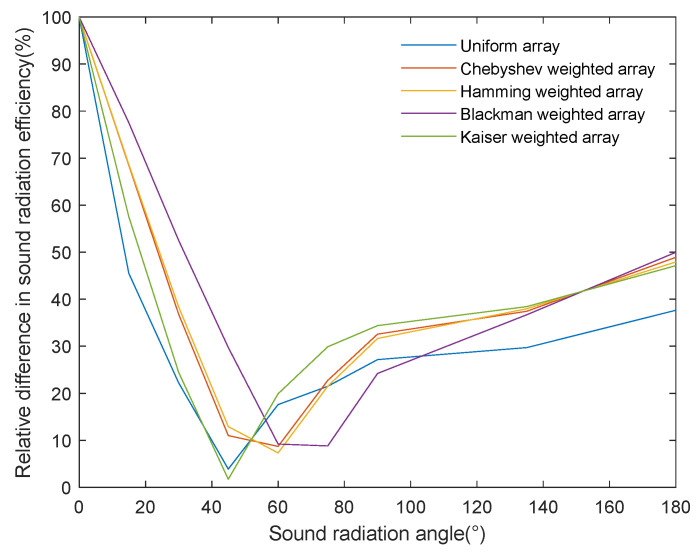
Relative difference between the proposed method and the half-power angle method in calculating the acoustic radiation efficiency.

In this study, the boundary element (BEM) was used as the method of numerical simulation. A three-dimensional geometric model of the underwater ultrasonic transducer array, including solid mechanics and electrical and acoustic models, was established using finite-element multiphysics field simulation software. By applying the piezoelectric effect and acoustic–solid coupling, the distributions of acoustic radiated sound pressure and acoustic radiated sound power were solved, and the data were post-processed and analyzed. Figure 4 shows the sound pressure level distribution of the underwater sound field of different arrays used as experimental sound sources to reflect spatial heterogeneity and further verify the effectiveness of the evaluation method in analyzing the radiation shape and radiation field distribution characteristics of the sound source. Compared with a uniform array, a weighted array performs more significantly in side-lobe suppression and out-of-band attenuation, and the sound field distribution also changes according to the weighted amplitude. The sharper the amplitude weighting, the more obvious its side-lobe suppression effect. Table 1 lists the parameters used in the reference model. As shown in Figure 4, a closed envelope sphere was set around the ultrasonic transducer array to obtain the sound radiation sound pressure distribution within the closed surface and the sound radiation angle. To more clearly observe and analyze the distribution characteristics of ultrasonic energy in space, we rotated some three-dimensional results to the x–z plane, which could more intuitively show the law behind the change in the sound pressure level on this plane and helped us better understand the radiation performance of ultrasonic energy. This is crucial for evaluating the sound energy radiation performance of underwater ultrasonic transducer arrays. Subsequently, the performance of the proposed method was compared and analyzed with the traditional and commonly used half-power angle evaluation method. The specific comparison results are detailed in Figure 5, Figure 6, Figure 7, Figure 8, Figure 9, Figure 10, Figure 11 and Figure 12.

## 4. Results and Discussion

Acoustic source radiation efficiency is an important parameter for evaluating the performance of underwater ultrasonic energy radiation, reflecting the utilization of acoustic radiation energy [40]. Acoustic source radiation power and radiation angle are important parameters in evaluating acoustic radiation efficiency [16]. Acoustic-radiated power is the acoustic energy radiated outward by a sound source in a unit of time, reflecting its radiation capacity [41]. The sound radiation angle describes the distribution of the sound energy radiated by a sound source in different directions, reflecting its directivity characteristics [42]. The more directional the source is, the more efficient the radiation is; this is because the acoustic energy is more centrally radiated in the desired direction rather than scattered in others [25]. As mentioned in the Introduction, the half-power angle evaluation method is a commonly used representative method for evaluating the radiation angle and power of a sound source. In order to more comprehensively evaluate the performance of our algorithm and verify its effectiveness and superiority in the evaluation of underwater ultrasonic energy radiation performance, the present method was compared with the half-power-angle evaluation method, considering the aspects discussed in the following subsections.

### 4.1. Acoustic Radiation Power

Figure 5 shows a comparison of the beam pattern of the uniform and Chebyshev-weighted array obtained based on the half-power angle method. The horizontal and vertical axes represent, respectively, the acoustic-radiated power angle and the array’s directivity. Compared to the uniform array, the Chebyshev-weighted array has a lower side-lobe level, which is conducive to suppressing energy leakage. The half-power angle method cannot directly obtain the maximum power of the main lobe, while the other power angle values are calculated based on the gain factor associated with the main lobe’s maximum. Therefore, the half-power angle method cannot directly calculate the sound power independently but only obtain the power ratio associated with the main lobe, ignoring the effect of the side-lobe energy, failing to fully consider the unevenness of the sound field distribution, and lacking precision in the calculation of acoustic-radiated power.

Figure 6 demonstrates the comparative results of the method proposed in this paper for calculating the acoustic-radiated power of a homogeneous ultrasonic transducer array and a Chebyshev-weighted array. The horizontal and vertical axes represent, respectively, different acoustic-radiated power angles and the acoustic-radiated power. The upper and lower sets of data curves in the figure demonstrate the variation in acoustic-radiated power with the acoustic radiation angle at different acoustic radiation radii. Among them, the upper set of data is the result of calculating the acoustic-radiated power of a uniform ultrasonic transducer array with the proposed method, while the lower set of data is the result of a Chebyshev-weighted array. Finite-element multiphysics field simulation software was used for the experimental validation of this method. Accurate meshing may affect the accurate analysis of acoustic pressure waves in water. The results may fluctuate when calculating a smaller acoustic radiation radius, but, as the acoustic radiation radius increases, the influence of the grid delineation accuracy lessens, and the data curves are basically consistent. This indicates that the acoustic-radiated power is an inherent property of sound source radiation, independent of the radiation distance. In addition, the acoustic-radiated power obtained via this method shows a monotonously increasing trend with the increase in the acoustic radiation angle, which indicates that it can objectively reflect the sound source’s radiation capability.

The half-power angle evaluation method calculates acoustic-radiated power through the main-lobe correlation value, ignoring the influence of the side-lobe energy; in contrast, the proposed method takes into full consideration the source’s radiation shape and the radiation field’s distribution characteristics, which makes the calculation results more accurate and objectively reflective of the source’s radiation capability.

### 4.2. Sound Power Analysis Based on a High-Resolution Spatial Distribution Model

In order to accurately characterize the spatial distribution of sound power, the sound field needs to be measured using hydrophones distributed at different locations. The signals obtained by each hydrophone can be used to calculate the sound pressure level and phase at the corresponding location. By combining the measurements from different hydrophones, a three-dimensional map of the acoustic field can be constructed to ensure the comprehensive coverage of the ultrasonic emitter’s radiation area.

The hydrophone layout should consider coverage and spatial resolution. Theoretically, a perfect spherical analysis of acoustic-radiated power should be based on the calculation of a complete sphere. However, in practice, it is not possible to measure the relevant acoustic parameters such as sound intensity and pressure within a certain area by densely arranging the acoustic instruments, and, because there is a certain spacing between the measuring instruments, a certain amount of error will inevitably occur in the actual measurement process. Figure 7 shows the simulation model established in this study in the three-dimensional water space defined by the x, y, and z axes. The model divides the complete spherical surface into grids and performs spherical-fitting calculations in grid units. This dense grid cell-fitting method can infinitely approximate the spherical surface, and, compared to the acoustic instrument measurement method in practical applications, its calculation accuracy is higher due to more measurement nodes and a higher spatial resolution of the acoustic field calculation measurement.

In the finite-element simulation model of this study, we divided the grid size according to the wavelength. Since the emission frequency of the acoustic source was fixed in this experiment, the grid cell size remained constant for different radii of acoustic power sphere radiation. However, as the radiation radius increased, the surface area of the acoustic radiation sphere increased, the number of grid cells increased accordingly, and the grid cell area was smaller relative to the surface area of the acoustic radiation sphere, which improved the computational accuracy. In this paper, the acoustic radiation field calculation results under an acoustic radiation radius of 300 cm have been used as the basis for the experiments, and the acoustic radiation performances of the uniform array and the Chebyshev-, Hamming-, Blackman-, and Kaiser-weighted arrays have been compared and analyzed.

### 4.3. Acoustic Radiation Angle

The angle of sound radiation refers to the distribution of the sound energy radiated by a sound source in different directions, reflecting the source’s directional and directivity characteristics. The more directional a sound source is, the more efficiently it radiates. This is due to the fact that the sound energy is radiated more centrally in the desired direction, rather than being scattered in others.

As shown in Figure 8, we compared the beam patterns of a uniform array based on the half-power angle method and an array processed by different weighting algorithms. On the horizontal axis, we show the acoustic radiation power angle, while, on the longitudinal axis, the array directivity is represented. The half-power angle method determines the beamwidth in a specific direction through the beam’s directivity pattern. However, while the weighted array effectively suppresses the side-lobe level, it also causes the main lobe’s beamwidth to increase compared to the uniform array. In order to more deeply evaluate the sound energy radiation performance, we needed to analyze the energy projected by the sound source in its corresponding direction after a certain transmission distance. The half-power angle method is based on the main lobe’s beamwidth within a specific sound radiation angle, takes attenuation as the benchmark, and can only rely on the beam gain to qualitatively analyze the radiated energy at the required sound radiation angle. However, it cannot quantitatively analyze the energy concentration at the corresponding sound radiation angle.

Therefore, this study proposes an underwater ultrasonic energy radiation performance evaluation method based on the spatial distribution characteristics of sound power, aiming to more effectively solve the performance evaluation problem of sound energy radiation concentration. As shown in Figure 9, this method can be used to compare the radiation power of different arrays at a radiation radius of 300 cm. The horizontal and vertical axes represent, respectively, the sound radiation power angle and power. This method uses Formula (20) to substitute the numerical values of specific directions and distances, calculates the effective radiation area sound power of the underwater ultrasonic transducer array according to derived Formulas (21) and (34), and uses Formula (35) to calculate the total energy radiated power. The results show that the total sound power of the weighted array is about 1 mw, compared to about 3 mw for the uniform array, showing that the former is slightly lower.

The method proposed in this paper can fully reflect the energy radiation concentration in the acoustic radiation field, obtain specific acoustic radiation power values according to the acoustic radiation angle, and reproduce the overall distribution of acoustic radiation energy and power as well as local details. In contrast, the half-power angle evaluation method can only obtain the beamwidth projected to a certain radiation angle and cannot independently calculate the exact acoustic-radiation-angle-specific power value.

### 4.4. Acoustic Radiation Efficiency

Sound radiation efficiency is the ratio of sound energy emitted by a sound source to the energy it consumes, which reflects the utilization of sound radiation energy.

As shown in Figure 10, the array energy share at different acoustic radiation power angles is calculated using the half-power angle method. The horizontal and vertical axes represent, respectively, the acoustic radiation power angle and the ratio of the radiated energy at different acoustic radiation angles to the radiated energy of the main lobe, i.e., the acoustic radiation efficiency. The efficiency derived from the half-power angle evaluation method is calculated based on the main lobe’s beam, which gradually decreases with the increase in the acoustic radiation angle and is also affected by the fluctuation in the side-lobe level. Undoubtedly, this result does not respect the objective laws of the acoustic energy radiation process, because it does not exist independently but is limited by the main lobe’s beam and lacks precision.

However, the radiation efficiency of the acoustic source is a key parameter for evaluating the performance of underwater ultrasonic wireless energy transmission. To effectively solve this problem, this paper proposes a method for evaluating the performance of underwater ultrasonic energy radiation based on the spatial distribution characteristics of acoustic power. As shown in Figure 11, this method calculates the comparative acoustic radiation efficiency results of different arrays, with the horizontal and vertical axes representing the acoustic radiation angle and efficiency, respectively. The acoustic radiation efficiency calculated using this method shows a monotonically increasing trend with the increase in the acoustic radiation angle. The curves for the different arrays’ data all converge to 50% at 180°, which is consistent with the fact that the acoustic radiation from the ultrasonic transducer arrays we have hereby studied is axisymmetric. The acoustic radiation efficiency calculated using this method is not constrained by the main lobe’s beam and can objectively reflect the degree of acoustic radiation energy utilization. In addition, we know that weighted arrays effectively suppress the energy leakage of the side lobe while, at the same time, leading to an increase in the width of the main lobe, i.e., a deterioration of the beam’s directivity. From the results calculated with this method, the energy radiation efficiency of the uniform array is higher than that of the weighted array, which indicates that the beam’s directivity has a more significant effect on the acoustic energy radiation efficiency compared to side-lobe suppression.

Figure 12 shows the degree of data deviation reflected by the absolute value of the difference between the acoustic radiation efficiency obtained by the half-power angle method and the method proposed in this paper, where the x and y axes represent, respectively, different acoustic radiation angles and the relative difference in the calculated acoustic radiation efficiency. To further evaluate the data calculation accuracy between the half-power angle method and the one proposed in this paper, Table 2 provides the root mean square error of the acoustic radiation efficiency calculated using the proposed method relative to the half-power angle method, reflecting the accuracy of the latter. The curve and table data show the relative accuracy of the calculated acoustic radiation efficiency, from high to low: Blackman-, Hamming-, Chebyshev-, and Kaiser-weighted arrays and uniform array. This order is directly related to the degree of side-lobe suppression in each array configuration, confirming that the calculation of acoustic power via the half-power angle method ignores the influence of side-lobe energy.

The new evaluation method proposed in this paper has proven its superiority in calculating the acoustic radiation efficiency. Our method, which is not constrained by the main lobe’s beam, was able to objectively reflect the degree of acoustic radiation energy utilization, and the acoustic radiation efficiency showed a monotonically increasing trend with the increase in the acoustic radiation angle. In addition, by applying the method proposed in this study to compare the acoustic radiation performance of uniform ultrasonic transducer arrays and different weighted arrays, we found that the uniform array’s energy radiation efficiency was higher than that of the weighted arrays, indicating that the effect of acoustic energy concentration on acoustic energy radiation efficiency is greater than that of side-lobe leakage energy. Therefore, in practical underwater wireless energy transmission applications, priority is given to the configuration of acoustic source parameters that accomplish high energy concentrations.

### 4.5. Analysis of the Influence of the Underwater Environment on the Acoustic Energy Radiation Efficiency

Based on the derived equations for underwater acoustic wave propagation in a complex impedance density medium influenced by multiple parameters, this study found that variations in temperature, salinity, and pressure in water typically result in concurrent changes in density and sound speed, with temperature having the most significant impact. Generally, higher water temperatures accelerate sound speed, as the increased molecular activity of water at higher temperatures speeds up sound wave propagation. An increase in sound speed usually indicates a change in the acoustic impedance of the water medium, which is the product of density and sound speed, affecting the reflection and transmission of sound waves. When sound speed increases while density remains constant, acoustic impedance rises, potentially reducing reflection at the interfaces between different media and increasing transmission, thereby enhancing the efficiency of sound propagation in water. However, in real environments, as temperature rises, water density decreases. Beyond approximately 20 °C, the rate of increase in sound speed begins to slow down due to the more pronounced effects of water compressibility.

**Figure 13:**
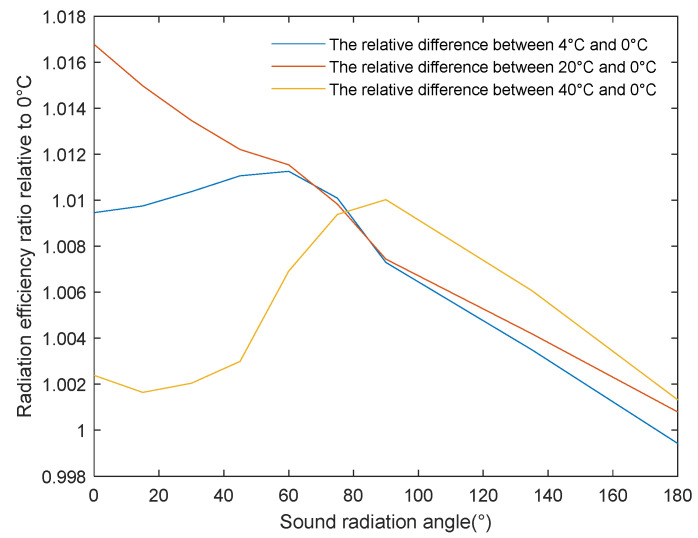
Comparison of the relative difference between the acoustic radiation efficiency in different water temperature environments and at the reference temperature.

This study selected a uniform array as a reference to analyze the impact of different temperatures on underwater acoustic radiation efficiency. As shown in Figure 13, to facilitate the observation of changes in the sound radiation efficiency at different temperatures, the sound radiation efficiency ratios at different temperatures relative to the reference temperature of 0 °C were calculated. The x and y axes represented, respectively, the sound radiation angle and the ratio of the sound radiation efficiency at different temperatures to that at 0 °C.

When the temperature rose from 0 °C to 4 °C, the relative ratio curve of the sound radiation efficiency was, overall, higher than 1, indicating that, when the temperature changes from low to high, the sound radiation efficiency first increases with the increase in temperature. In the temperature range from about 4 °C to 20 °C, the acoustic radiation efficiency gradually increased, but the rate of increase decreased as the temperature further increased. When the temperature exceeded 20 °C and reached 40 °C, the acoustic radiation efficiency increased compared to 0 °C but decreased compared to 4 °C and 20 °C. This was because the continued increase in temperature also affected the thermal conductivity of water, and the heat exchange during sound wave propagation led to energy loss. However, when the sound radiation angle exceeded 80°, the increase in sound radiation efficiency became more significant. This was because the increase in sound speed meant that the sound waves could propagate over longer distances without significant attenuation, thereby increasing the effective propagation distance of sound power and expanding the coverage area of the sound source.

This research, utilizing high-resolution spatial distribution data to analyze the impact of temperature changes on sound wave propagation, confirmed that the method could accurately predict sound radiation power and effectively reflect the dynamic changes in sound field distribution.

## 5. Conclusions

In this study, we propose a method for evaluating the performance of underwater ultrasonic energy radiation based on the spatial distribution characteristics of acoustic power. By modeling the acoustic power distribution in complex impedance–density aqueous media and combining numerical simulation and experimental validation methods, the spatial variation of acoustic power and its influence on the energy transfer efficiency are studied in depth. The results show that compared with the traditional method, the evaluation method proposed in this paper is more accurate for the calculation results of acoustic energy radiation, which provides an important theoretical basis and practical guidance for the optimization design and performance improvement of underwater ultrasonic wireless energy transmission system. The main conclusions are as follows.

The evaluation method based on the spatial distribution characteristics of acoustic power proposed in this paper can comprehensively reflect the energy distribution and spatial heterogeneity of sound source radiation. Compared with the traditional method, the method significantly improves the accuracy of the calculation results of acoustic energy radiation.

In the acoustic-radiated efficiency evaluation, the method in this paper significantly outperforms the traditional half-power angle method. The proposed method in this paper is able to calculate the acoustic-radiated power at a specific angle independently, which avoids the shortcomings of the traditional method in the calculation of the side lobe energy and provides a more accurate acoustic-radiated efficiency evaluation.

In this study, the effect of temperature changes in the acoustic field on acoustic wave propagation is analyzed by using high-resolution spatial distribution data. Combined with numerical simulation and experimental validation, the data results obtained from this method not only verify its ability to accurately predict the acoustic-radiated power, but also effectively reflect the dynamic changes of the sound field distribution.

In summary, the method proposed in this paper shows obvious superiority in acoustic radiation performance evaluation and provides an important reference for the study of underwater ultrasonic energy radiation performance.

## Figures and Tables

**Table 1:** Model parameters.

Symbol	Model Parameter	Parameter Value
$V_{water}$	Volume of free-space water	2 × 2 × 4 m
*N*	Number of array elements	8
*a*	Radius of piezoelectric ring	1 cm
*h*	Thickness of piezoelectric ring	1 mm
*d*	Array element spacing	4 cm
*T*	Temperature of water medium	277.15 K, 293.15 K, and 313.15 K
$T_{ref}$	Reference temperature of water medium	273.15 K
$S_{p}$	Salinity of water medium	35
*pH*	pH of water medium	8
$V_{rms}$	Rated drive voltage	10 V
$d_{33}$	Piezoelectric constant	500 pC/N
$R_{radiation}$	Radiation power sphere radius	15, 25, 35, 45, 75, 100, 150, 200, 250, and 300 cm

**Table 2:** The accuracy of the method proposed in this paper in calculating the acoustic radiation efficiency relative to the half-power angle method.

Array Type	Relative Accuracy
Uniform array	42.6721%
Chebyshev-weighted array	49.0047%
Hamming-weighted array	49.0052%
Blackman-weighted array	51.9048%
Kaiser-weighted array	47.2426%

## Data Availability

Data are contained in within the article.
